# Anti-Neoplastic Activity of Estrogen Receptor Beta in Chemoresistant Triple-Negative Breast Cancer

**DOI:** 10.3390/cancers17132132

**Published:** 2025-06-25

**Authors:** Xiyin Wang, Michael J. Emch, Matthew P. Goetz, John R. Hawse

**Affiliations:** 1 Graduate School of Biomedical Sciences, Mayo Clinic, Rochester, MN 55905, USA; wang.xiyin@mayo.edu; 2 Department of Oncological Sciences, University of Utah School of Medicine, Salt Lake City, UT 84132, USA; michael.emch@hci.utah.edu; 3 Department of Oncology, Mayo Clinic, Rochester, MN 55905, USA; goetz.matthew@mayo.edu; 4 Department of Biochemistry and Molecular Biology, Mayo Clinic, Rochester, MN 55905, USA

**Keywords:** triple-negative breast cancer, estrogen receptor beta, chemotherapy, chemotherapy resistance, paclitaxel, doxorubicin

## Abstract

Background: Targeted therapies, such as endocrine agents, have significantly improved outcomes for patients with estrogen receptor alpha-positive (ERα+) breast cancer. Unfortunately, for patients with triple-negative breast cancer (TNBC), which lack expression of ERα and HER2, there remains a dearth of targeted adjuvant agents. We discovered that estrogen receptor beta (ERβ) is expressed in approximately 20% of TNBC cases, and its activation has been shown to inhibit proliferation, invasion, and migration in preclinical models. However, it remains unclear whether ERβ-targeted therapies maintain efficacy following the development of chemoresistance. Methods: To address this question, we generated ERβ+ TNBC cell line models with acquired resistance to paclitaxel or doxorubicin. We then assessed their response to ERβ-targeted therapies and analyzed transcriptomic changes associated with chemoresistance and ERβ ligand treatment. Results: Chemotherapy-resistant ERβ+ TNBC cells retained sensitivity to ERβ-targeted therapies and, in some cases, exhibited enhanced responsiveness. ERβ expression did not compromise chemotherapy efficacy in treatment-naïve cells. Chemotherapy-resistant cells had a vastly altered transcriptome and surprisingly, a heavily reduced ERβ transcriptome, compared to sensitive cells despite the maintenance of ERβ-driven anti-neoplastic activity. Conclusions: These findings suggest that ERβ remains a relevant drug target in chemotherapy-refractory disease and has aided in the refinement of a minimal ERβ transcriptomic signature associated with response to ERβ-targeting agents, further informing the primary mechanisms through which ERβ elicits its tumor suppressive effects.

## Simple Summary

The standard of care for triple-negative breast cancer is chemotherapy followed by surgical resection, and in most cases, additional rounds of chemotherapy treatment. While highly effective, approximately half of patients will experience disease recurrence that does not respond to additional rounds of treatment. Previous findings demonstrate that estrogen receptor beta (ERβ) is expressed in upwards of 20% of triple-negative tumors, and that ERβ functions as a tumor suppressor in triple-negative breast cancer models, it was of interest to determine if the tumor-suppressive effects of ERβ persisted in chemotherapy-resistant forms of the disease. Here, we demonstrate that the activation of ERβ in chemotherapy-resistant triple-negative breast cancer cell lines elicits equivalent or superior inhibitory effects compared to chemotherapy-sensitive counterparts. These findings support the potential use of ERβ-targeting agents in advanced forms of the disease.

## 1. Introduction

Triple-negative breast cancer (TNBC) is a heterogeneous group of tumors defined by the lack of estrogen receptor alpha (ERα) and progesterone receptor expression, as well as the absence of human epidermal growth factor receptor 2 (HER2) amplification or overexpression. TNBC accounts for 15–20% of all breast tumors and has the worst prognosis of the various breast cancer subtypes [1]. The reference treatment for early-stage TNBC remains an anthracycline- and taxane-based neoadjuvant chemotherapy regimen, often including a checkpoint inhibitor such as pembrolizumab, followed by surgery [2]. In advanced/metastatic disease, most patients will receive sequential chemotherapy regimens (in combination with checkpoint inhibitor therapy for tumors that are PDL1-positive) or combination chemotherapy in the case of rapid progression [3,4].

Anthracyclines, such as doxorubicin, function through intercalation with DNA and inhibition of topoisomerase II, preventing transcription, arresting cells in the G1/G2 phase of the cell cycle, and inducing apoptosis [5]. Taxanes, such as paclitaxel, function at high concentrations by preventing the depolymerization of microtubules, leading to mitotic catastrophe and apoptosis during the M phase. At lower concentrations, they function by increasing the length of mitosis and the rate of mitotic error leading to aneuploidy and eventual cell death [6]. Combinatorial chemotherapy regimens combine agents with distinct mechanisms of action for improved anti-neoplastic effects, but they come at the cost of increased side effects, some of which may be severe [7]. An estimated 50% of TNBC patients achieve a pathologic complete response following neoadjuvant chemotherapy (NAC) combined with checkpoint inhibitor therapy highlighting the fact that de novo and acquired chemotherapy resistance is frequent [8]. Complicating the clinical management of TNBC further is the lack of effective adjuvant therapies for those with residual disease following NAC and the dearth of agents with meaningful activity in the metastatic setting. Indeed, chemotherapy resistance accounts for 90% of therapy failure across cancers, and in TNBC, disease recurrence and progression typically occur within 3–5 years of initial diagnosis [9,10]. As such, it is of continued importance to identify and develop therapeutic approaches for these patients.

Estrogen receptor beta (ERβ), encoded by the *ESR2* gene, is a steroid nuclear hormone receptor expressed in up to 20% of TN breast tumors [11]. As a transcription factor, ERβ plays a central role in the regulation of genes and the response of cells to estrogen [12]. ERβ expression is abundant in normal breast epithelium, but is lost in most breast tumors, supporting its function as a tumor suppressor in the breast [13,14]. Activation of ERβ by 17-β estradiol (E2), or ERβ selective agonists, has been shown to elicit anti-neoplastic activity in TNBC, both in vitro and in vivo [15,16,17,18]. ERβ confers these effects in part through suppression of NFκB/p65, wherein it repurposes EZH2 to epigenetically silence oncogenic p65 signaling [12]. Despite these discoveries and advances, the relevance of ERβ as an effective target following disease recurrence and chemotherapy resistance has not been determined.

Here, we describe the development and characterization of chemotherapy-resistant models of ERβ-positive TNBC and evaluate how chemotherapy resistance impacts the role of ERβ as a tumor suppressor. Further, we delineate the ERβ transcriptomes in chemotherapy-sensitive vs. chemotherapy-resistant isogenic models and determine if ERβ expression impacts responsiveness to DNA-damaging and microtubule-inhibiting agents. Our findings demonstrate that the anti-neoplastic properties of ERβ-targeted therapies remain effective in the setting of chemotherapy resistance and demonstrate that ERβ expression does not interfere with the efficacy of chemotherapy, further supporting the utility of ERβ-targeting drugs in newly diagnosed and advanced forms of TNBC.

## 2. Materials and Methods

### 2.1. Reagents

Doxorubicin was purchased from Pfizer (New York, NY, USA) and paclitaxel from Actavis (Dublin, Ireland). Doxycycline, 17-β estradiol (E2), and dexamethasone were purchased from Sigma Aldrich (St. Louis, MO, USA). The ERβ agonist LY500307, ifosfamide, carmustine, pantoprazole, and lansoprazole were purchased from Cayman Chemical Company (Ann Arbor, MI, USA). Cisplatin was purchased from Fresenius Kabi USA (Lake Zurich, IL, USA).

### 2.2. Cell Culture

Doxycycline-inducible TNBC MDA-MB-231-ERβ and MDA-MB-468-ERβ cell lines were generated as previously described [15,18]. Cells were maintained in phenol red-free DMEM/F12 medium (Jango Bio, Fitchburg, WI, USA) supplemented with 10% FBS (Gemini Bioproducts, Sacramento, CA, USA), 5 mg/L blasticidin S (Roche Applied Science, Indianapolis, IN, USA), 500 mg/L zeocin (Invitrogen, Thermo-Fisher Scientific, Waltham, MA, USA), and 1% antibiotic-antimycotic (Gibco, Thermo-Fisher Scientific, Waltham, MA, USA). To induce ERβ expression, cells were continuously treated with 100 ng/mL doxycycline, with the treatment duration specified for each experiment.

### 2.3. Generation of Chemotherapy-Resistant Lines

Chemotherapy-resistant isogenic cell lines were developed independently for doxorubicin and paclitaxel using 10–20 cycles of pulsed chemotherapy administration as described previously [19,20,21]. Cells were first treated with low-dose chemotherapy for 2 h, followed by a medium change into the standard chemotherapy-free medium. The cells were then allowed to grow until the surviving fraction of cells were proliferating in log phase. Once the cell population neared confluence, another pulse of chemotherapy was administered for 2 h. This process was repeated with increasing drug concentrations until the IC50 of each cell line had risen by at least 3–6-fold for a given drug. Parental cells were maintained and passaged in parallel with the resistant cells throughout the selection period.

### 2.4. RT-PCR

Total RNA was isolated using Trizol reagent (Invitrogen, Thermo-Fisher Scientific, Waltham, MA, USA) following the manufacturer’s protocol. One microgram of RNA was reverse transcribed using the iScript™ cDNA Synthesis Kit (Bio-Rad Laboratories, Hercules, CA, USA). Quantitative PCR was conducted using PerfeCTa™ SYBR Green Fast Mix™ (Quanta Biosciences, Gaithersburg, MD, USA) and a Bio-Rad CFX Real-Time PCR detection system. Gene expression levels were determined using the ΔΔCT method with HPRT1 as a control. Primers were purchased from Integrated DNA Technologies (IDT, Coralville, IA, USA) and their sequences are indicated in Supplemental Table S1.

### 2.5. Western Blotting

Cells were lysed in NETN buffer (150 mM NaCl, 1 mM EDTA, 20 mM pH 8.0 Tris, 0.5% NP-40) containing 1x cOmplete™ Protease Inhibitor Cocktail without EDTA (Roche Applied Science, Indianapolis, IN, USA) and 1x PhosSTOP™ phosphatase inhibitor (Roche Applied Science, Indianapolis, IN, USA). Protein concentrations were determined using a Pierce BCA protein assay kit (Thermo Scientific, Waltham, MA, USA). Fifty µg of total protein lysate was mixed with SDS-PAGE loading buffer (60 mM Tris pH 8.0, 2% SDS, 5% Glycerol, 0.01% Bromophenol blue, 2% Beta-mercaptoethanol), boiled for 5 min, and separated on SurePAGE Bis-Tris 4–12% gradient gels (GeneScript, Piscataway, NJ, USA) at 70 mA for 1 h. Protein was transfered to Immobilon^®^-FL PVDF membranes (MilliporeSigma, Burlington, MA, USA) using a cooled cassette at 550 mA for 30 min. Proteins of interest were detected using an iBind automated western system (Invitrogen, Thermo-Fisher Scientific, Waltham, MA, USA) following the manufacturer’s instructions. Bands were visualized using the Odyssey Fc system (LI-COR, Lincoln, NE, USA), with imaging in the 600 nm channel (30 s), 700 nm channel (2 min), and 800 nm channel (2 min) for ladder and protein detection. The antibodies utilized were as follows: ERβ (PP-PPZ0506-00, mouse monoclonal, Perseus Proteomics, Tokyo, Japan) at a 1:500 dilution and vinculin (ab155120, rabbit polyclonal, Abcam, Waltham, MA, USA) at a 1:2000 dilution. Fluorescent secondary antibodies, IRDye (LI-COR, Lincoln, NE, USA) IR 680 RD and 800 CW, were used at 1:2000 for all blots.

### 2.6. Proliferation Assays

One thousand cells per well were plated in 96-well tissue culture plates (Corning, Glendale, AZ, USA) using 10% charcoal-stripped FBS (GE Healthcare Life Sciences, Pittsburg, PA, USA) containing DMEM-F12 medium (Gibco, Thermo-Fisher Scientific, Waltham, MA, USA) with 100 ng/mL doxycycline added as indicated. Twenty-four hours later, the medium was removed and replaced with medium containing the respective drug treatments +/− doxycycline. After 7 days, proliferation was measured using crystal violet (Millipore-Sigma, Burlington, MA, USA) assays. Briefly, 25 μL/100 μL of 25% *v/v* glutaraldehyde (Millipore-Sigma, Burlington, MA, USA) was added to each well and the plates were rocked for 10 min at room temperature. Cell culture medium and glutaraldehyde were removed, and plates were rinsed under running distilled water 4–5 times after which 50 μL of crystal violet staining solution (0.5 g crystal violet, 25 mL methanol, and 75 mL water) was added to each well followed by rocking for an additional 10 min. The staining solution was removed, and plates were rinsed thoroughly under running distilled water and allowed to dry overnight. The following day, crystal violet was solubilized by the addition of 100 μL of 1:1 100 nM sodium citrate and 100% ethanol solution to each well. Following 10 min of rocking, the absorbance at 562 nm was recorded using a Spectramax M3 instrument (Molecular Devices, San Jose, CA, USA).

### 2.7. Karyotyping Analysis

Karyotyping was performed within the Mayo Clinic Cytogenetics Laboratory following standard procedures. Briefly, parental, D-R, and P-R cells were arrested in metaphase and fixed using a mixture of methanol and acetic acid. Slides were prepared by dropping a suspension of fixed cells onto glass slides followed by drying. Slides were aged and subsequently banded using trypsin and Giemsa stain. Chromosome analysis was performed by a cytogeneticist to identify numerical and structural abnormalities. A total of 20 metaphase spreads were analyzed for each of the three cell lines. Anomalies were defined as two or more cells with the same numerical or structural abnormalities. The abnormalities were visualized using web-based CytoConverter (https://jxw773.shinyapps.io/Cytogenetic__software/) [22].

### 2.8. Migration Assays

For migration assays, cells were pre-treated with doxycycline and respective treatments for 3 days prior to plating. Twenty-five thousand pre-treated cells per well were plated in 96-well ImageLock™ tissue culture plates (Essen BioScience, Ann Arbor, MI, USA) and allowed to adhere overnight and proliferate for 2 additional days prior to scratching. Scratches were made using the 96-pin wound-making tool (Essen Bioscience, Ann Arbor, MI, USA). Plates were transferred to an IncuCyte S3 instrument (Sartorius, Göttingen, Germany) and imaged every 2 h for 72 h.

### 2.9. Invasion Assays

Matrigel (Corning, #356231, Glendale, AZ, USA) was thawed and diluted to 100 μg/mL in cell culture medium. Next, 50 μL of Matrigel-containing medium was dispersed into each well of 96-well ImageLock™ plates followed by incubation at 37 °C overnight. The next day, the medium was discarded, and 25,000 pre-treated cells were plated in 100 μL of medium and allowed to adhere for 4 h. Then, 50 μL of 8 mg/mL matrigel containing medium was added to each well just prior to the scratch. Plates were then scratched using the 96-pin WoundMaker tool (Essen Bioscience, Ann Arbor, MI, USA). After 5 min, an additional 50 μL of matrigel-containing medium was added and the plate was allowed to incubate at 37 °C for 30 min. An additional 100 μL of culture medium with the indicated treatments was added and the plates were transferred to the IncuCyte S3 instrument (Sartorius, Göttingen, Germany) and imaged every 2 h.

### 2.10. RNAseq

Cells were plated in triplicate in charcoal-stripped FBS containing medium plus 100 ng/mL doxycycline to induce ERβ expression. After 24 h, a medium change was performed containing doxycycline plus ethanol vehicle or 1nM E2. Twenty-four hours post-treatment, total RNA was isolated using the Qiagen miRNeasy mini kit (Qiagen, Hilden, Germany) following the manufacturer’s protocol. Library preparation was conducted using the TruSeq Stranded mRNA Library Prep Kit with TruSeq RNA Single Index Set B (Illumina, San Diego, CA, USA). Sequencing was performed by the Mayo Clinic Genome Analysis Core using an Illumina HiSeq 4000 platform to generate 50 base pair paired-end reads with approximately 50 million total fragment reads per sample. Sequencing quality was checked using FastQC (version 0.11.8). Reads were aligned to the GRCh38 (hg38) genome using STAR (version 2.7.3a) with soft-clipping enabled for adapter sequences. featureCounts (version 2.8.2) was used to assign mapped reads. Lowly expressed genes (RPKM ≤ 1 in all samples) were removed prior to analysis. Differential expression analysis was performed using edgeR (version 3.36.0) and significance was defined as |fold change| ≥ 1.5, *p* < 0.05 and FDR < 0.1.

### 2.11. Pathway Analysis

Gene Set Enrichment Analysis (GSEA) was performed using the list of genes determined to be regulated by E2 in parental and chemotherapy-resistant MDA-MB-231-ERβ cells to identify significant associations with other gene sets derived from the Kyoto Encyclopedia of Genes and Genomes (KEGG) and Gene Ontology (GO) databases, including Biological Process, Cellular Component, and Molecular Function. The number of permutations was set to 2000, and gene set size filters were applied to include sets containing between 15 and 500 genes. Normalized enrichment scores (NES) were used to determine significant associations. Additionally, E2-regulated gene sets were analyzed using Enrichr-KG, with four libraries selected (GO Biological Process, Reactome, KEGG, WikiPathway). Finally, E2-regulated pathways and predicted upstream regulators were determined using Ingenuity Pathway Analysis (IPA) with significance defined as *p* < 0.05 and |z-score| ≥ 2.

### 2.12. Mergeomics Analysis

Compound prediction analysis was performed using the Mergeomics 3.0 web-based platform (https://mergeomics.research.idre.ucla.edu/home.php), which implements the PharmOmics pipeline for drug repurposing based on gene expression signatures [23]. For each of the parental, D-R and P-R cell line models, upregulated and downregulated genes (|fold change| ≥ 1.5, *p* < 0.05 and FDR < 0.1) following E2 treatment were uploaded to the platform. The PharmOmics Pipeline was used to compare input gene sets with a curated reference database of drug-induced transcriptional responses to identify compounds with overlapping gene expression patterns. Predicted compounds for each gene set were compared, and compounds were prioritized based on their inclusion across all three cell line models. Compounds identified by this analysis were functionally evaluated for their ability to inhibit chemotherapy-sensitive and -resistant cell lines in an ERβ-independent manner.

### 2.13. Statistical Analyses

Except for the RNAseq studies, all experiments were conducted in technical replicates of 3–8 with a minimum of 3 independent biological replicates. Unless otherwise noted, all statistical analyses were performed using GraphPad Prism software (Version 10.5.0) with 1- or 2-way ANOVA and post hoc correction as appropriate. The number of replicates for each assay is specified throughout the figure legends, and *p*-values are denoted. Graphical summaries of the data were generated from one representative biological replicate and depict the mean and standard error of the mean across technical replicates.

## 3. Results

### 3.1. Generation and Characterization of Chemotherapy-Resistant ERβ+ TNBC Cell Lines

**Figure 1:**
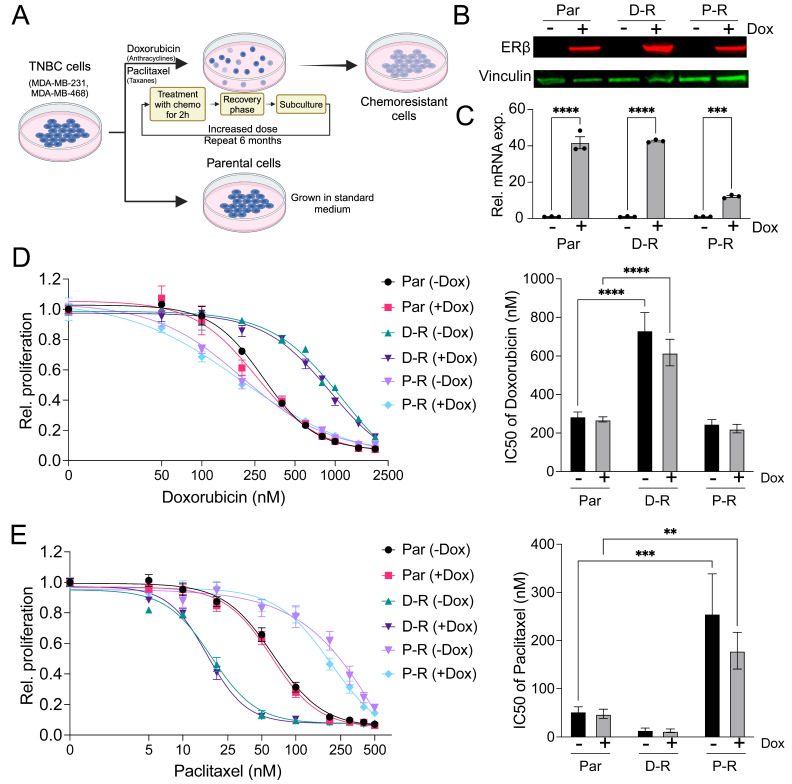
Generation of chemotherapy-resistant cell lines: (**A**) Schematic of the pulse-recovery approach used for developing chemotherapy-resistant cells. The figure was created with BioRender.com. Expression of ERβ protein (**B**) and mRNA (**C**) across parental and resistant MDA-MB-231 cell lines. Graphs depict mean ± SEM of triplicate measurements. Proliferative response of each MDA-MB-231 cell line to paclitaxel (**D**) or doxorubicin (**E**) administration with and without ERβ expression. IC50 values are shown to the right. The data shown are representative of 3–8 technical replicates derived from one of three independent experiments. Significance was determined by 2-way ANOVA. ** *p* < 0.01, *** *p* < 0.001, **** *p* < 0.0001.

To generate acquired resistance to doxorubicin and paclitaxel, we employed a pulse-based dose escalation method to avoid the pitfalls of chronic drug administration and to better mimic chemotherapy administration in patients [19]. MDA-MB-231-ERβ cells were initially treated for 2 h with 50 nM doxorubicin or 5 nM paclitaxel followed by a recovery period of 3–5 days and removal of dead cells. After 6 months of dose escalation following the same treatment strategy, doxorubicin-resistant (D-R) and paclitaxel-resistant (P-R) isogenic cell lines were derived (Figure 1A). Both resistant cell lines were shown to maintain doxycycline-inducible expression of ERβ at the protein (Figure 1B) and RNA level (Figure 1C).

Parental MDA-MB-231-ERβ cells exhibited IC50s of approximately 300 nM and 50 nM for doxorubicin and paclitaxel, respectively, regardless of ERβ expression (Figure 1D,E). D-R and P-R cells exhibited IC50s that were 3–6-fold higher than parental controls which were also independent of ERβ expression (Figure 1D,E). Notably, D-R cells were more sensitive to paclitaxel compared to parental controls, while the P-R cells exhibited no changes in sensitivity to doxorubicin (Figure 1D,E). Findings using the MDA-MB-468-ERβ cells were similar (Supplemental Figure S1).

To determine if gross chromosomal changes occurred during the establishment of chemotherapy resistance, we performed karyotyping on MDA-MB-231-ERβ parental cells and their isogenic D-R and P-R models using classical methods of chromosome visualization (Supplemental Figure S2). All metaphases were complex with similar ploidy levels and a few numeric and structural abnormalities were observed. Deletions or structural abnormalities in 6q were commonly observed across all cell lines as well as an 8p deletion and additional chromatin beyond 8q22 or 8q24.1 (Supplemental Figure S2). Furthermore, all lines showed 9p deletions and structural abnormalities in chromosome 15 with unidentifiable chromatin beyond band p11.2. D-R and P-R cells were vastly similar to the parental MDA-MB-231-ERβ cell line with the exception of numerical increases in chromosomes 7 and 17 (Supplemental Figure S2). These data reveal that prolonged exposure to increasing concentrations of doxorubicin and paclitaxel during the establishment of chemotherapy resistance did not lead to substantial alterations in the karyotype of these models. This observation is consistent with previous reports using matched tumor specimens prior to and following chemotherapy treatment of TNBC patients [24,25].

### 3.2. Effect of Chemotherapy Resistance on Responsiveness to ERβ-Targeted Therapy

**Figure 2:**
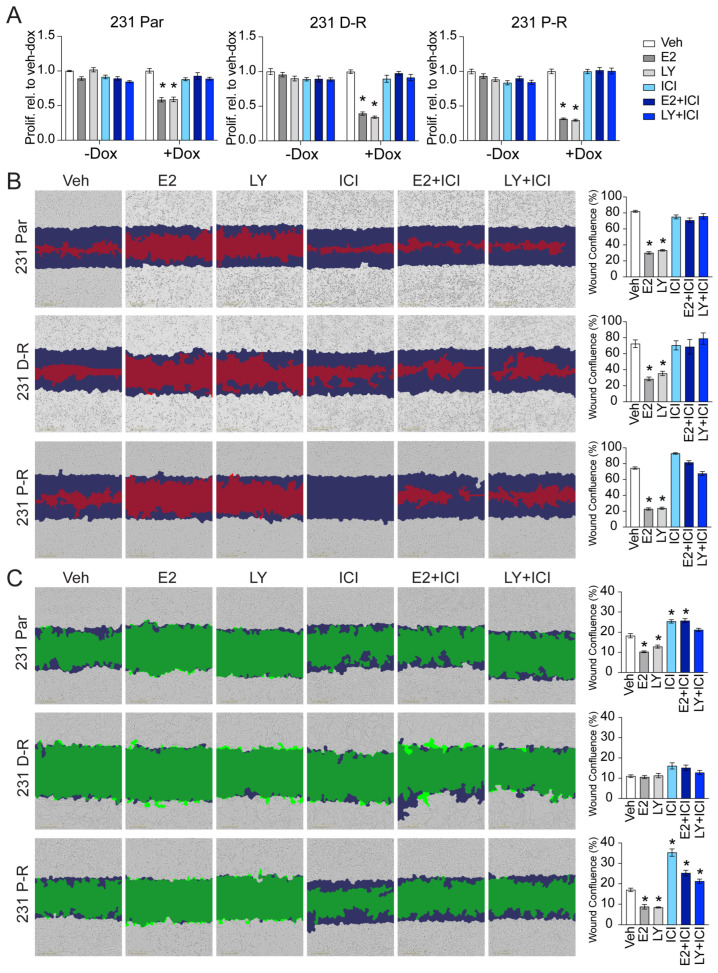
The activity of ERβ-targeted therapies in chemotherapy-resistant TNBC cells. (**A**) Proliferation rates of chemotherapy-sensitive and -resistant MDA-MB-231-ERβ cells in response to the ERβ agonists, E2 and LY, as well as the SERD ICI, alone and in combination. Migration (**B**) and invasion (**C**) of parental and chemotherapy-resistant cells 72 and 108 h after wound creation, respectively. Blue represents the initial scratch wound devoid of cells while red/green depicts the remaining wound area not occupied by cells at the conclusion of the experiment. Data shown are representative of 8 technical replicates derived from one of three independent experiments. Significance was determined by one-way ANOVA. * *p* < 0.05.

Following the confirmation of chemotherapy resistance and maintenance of ERβ expression, we next profiled the efficacy of ERβ-targeted therapies in the chemo-refractory setting. In parental MDA-MB-231-ERβ cells, treatment with E2 (1 nM) or the ERβ selective agonist LY500307 (LY; 10 nM) suppressed proliferation only when ERβ was expressed, an effect which was blocked by the addition of fulvestrant (ICI; 100 nM), a potent selective estrogen receptor degrader (SERD) that inhibits ERβ activity (Figure 2A). In D-R and P-R isogenic cell lines, treatment with E2 and LY potently suppressed proliferation, again only when ERβ was expressed, and treatment with ICI was sufficient to completely prevent these effects. Of note, the magnitude of ERβ-mediated suppression of cell proliferation was greater in chemotherapy-resistant cells compared to parental controls (Figure 2A).

In chemotherapy-sensitive MDA-MB-231-ERβ cells, treatment with E2 and LY reduced the cells’ migratory capacity (Figure 2B). Similarly, the activation of ERβ strongly inhibited migration of the ERβ+ chemotherapy-resistant models, effects that were attenuated in the presence of ICI (Figure 2B). Likewise, we profiled the invasive capacity of these models in response to ERβ agonists and antagonists. Under vehicle-treated conditions, the D-R cells exhibited little to no invasive ability over the timeframe of the experiment compared to the parental and P-R cells (Figure 2C). E2 and LY inhibited invasion of the parental and P-R cells. Notably, ICI increased the invasive capacity of chemotherapy-sensitive and -resistant cells alone, and in the presence of E2 or LY (Figure 2C). Taken together, these findings demonstrate that the anti-neoplastic activity of ERβ is maintained in the setting of chemotherapy resistance.

### 3.3. Transcriptomic Profiles of Chemotherapy-Resistant ERβ+ TNBC

**Figure 3:**
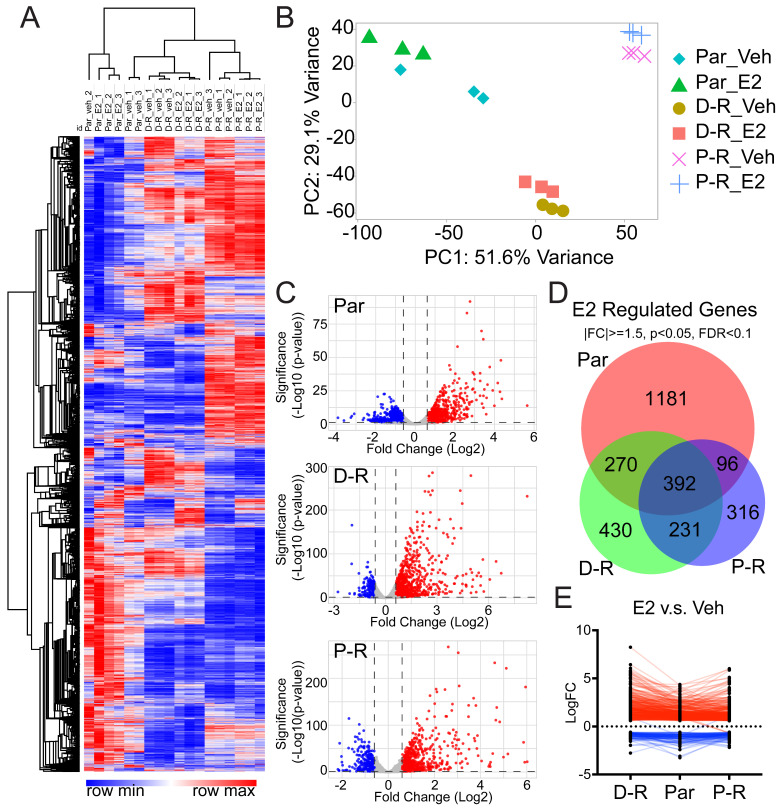
Basal- and E2-regulated transcriptomic profiles of parental and chemotherapy-resistant MDA-MB-231-ERβ cells. (**A**) Clustering and heatmap analysis of vehicle- and E2-treated MDA-MB-231-ERβ parental, D-R and P-R cell lines. The hierarchical clustered heatmap was generated using one minus Pearson correlation and average linkage. Each column represents independent samples while each row corresponds to a single gene. Expression levels are color-coded with red indicating high gene expression and blue indicating low gene expression. (**B**) A principal component analysis (PCA) plot depicting each cell line, treatment, and replicate sample. PC1 accounts for 51.6% of the variance, while PC2 accounts for 29.1%, highlighting distinct clustering of the parental and resistant cell lines and further separation based upon treatment. (**C**) Volcano plots for each cell line indicating genes found to be significantly induced (red dots) or repressed (blue dots) in response to E2 treatment (|FC| ≥ 1.5, *p* < 0.05, FDR < 0.1). (**D**) Venn diagram showing overlap of differentially expressed genes in the three cell lines in response to E2 treatment. (**E**) Line graph demonstrating the direction of the 392 genes identified to be regulated by E2 in all three cell lines. Nearly all of these genes were found to be regulated in the same direction (either E2-induced or -repressed) across all models.

To compare the basal gene expression profiles of parental MDA-MB-231-ERβ cells to their isogenic D-R/P-R derivatives, and to delineate the ERβ transcriptome in each of these models, RNA-seq was conducted following 24 h of treatment with vehicle or 1 nM E2. As expected, acquired therapeutic resistance conferred distinct transcriptional profiles in D-R and P-R cells compared to parental controls (Figure 3A,B). A total of 1745 and 2802 genes were found to be differentially expressed in D-R and P-R cells respectively, of which 514 and 380 were commonly up- and downregulated in both chemotherapy-resistant models (Supplemental Figure S3A,B).

In response to E2 treatment, 1939, 1323, and 1035 genes were differentially expressed in parental, D-R, and P-R cells respectively, of which 392 genes were commonly regulated in all 3 models (Figure 3C,D). When considering the direction of E2 regulation, 827, 1035 and 686 genes were upregulated and 1112, 288, and 349 genes were downregulated in the parental, D-R, and P-R models respectively (Supplemental Figure S3E,F). Among the E2-induced genes, 301 were commonly upregulated across all three cell lines while 87 genes were commonly downregulated across all three models (Supplemental Figure S3E,F). In total, 4 of the 392 commonly regulated genes (CCN2, LPAR1, NRCAM, LFNG) exhibited discordant regulation patterns between the three models (Figure 3E). Taken together, these results indicate that chemotherapy resistance and ERβ activation confer distinct and largely non-overlapping transcriptomic profiles, highlighting the context-specific regulation of gene expression in parental, D-R, and P-R models. However, they also revealed a core set of ERβ-regulated genes that are conserved across all models and which represent a minimal ERβ signature that may primarily mediate the anti-neoplastic activity of ERβ in TNBC.

### 3.4. Pathway Analysis of E2-Regulated Genes in Chemotherapy-Resistant ERβ+ TNBC Cells

**Figure 4:**
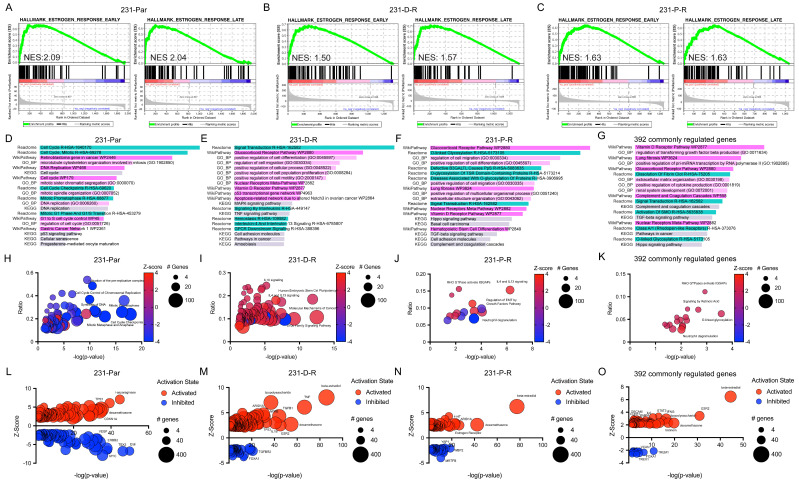
Pathway analysis of E2-regulated genes in MDA-MB-231-ERβ parental and resistant cell lines. (**A**–**C**) Gene Set Enrichment Analysis (GSEA) for hallmark early and late estrogen response pathways in Par (**A**), D-R (**B**), and P-R (**C**) cells. Normalized enrichment scores (NES) are shown for each pathway in each cell line, indicating robust estrogen pathway activation in all models, with the strongest enrichment observed in parental cells. (**D**–**G**) Pathway enrichment analysis using Enrichr-KG of E2-regulated genes in Par (**D**), D-R (**E**), and P-R (**F**) cell lines, as well as for the 392 commonly regulated genes in all cell lines (**G**) across four gene set libraries. Blue shading represents Reactome pathways, purple shading represents WikiPathway pathways, pink shading represents GO Biological Process (GO BP) pathways, and green shading represents KEGG pathways. Bar length represents −log (*p*-value), with longer bars indicating greater statistical significance. Pathways are ranked based on significance. (**H**–**K**) Pathway analysis using IPA of differentially expressed genes in response to E2 treatment in Par (**H**), D-R (**I**), and P-R (**J**) cell lines, as well as for the 392 commonly regulated genes (**K**), with significance defined as *p* < 0.05 and |z-score| ≥ 2. The *x*-axis represents −log (*p*-value) of pathway enrichment, while the *y*-axis represents the ratio of differentially expressed genes in the pathway relative to the total genes in the pathway. The size of each bubble corresponds to the number of genes in the pathway, and the color reflects the z-score, indicating activation (red) or inhibition (blue). (**L**–**O**) Upstream regulator analysis using IPA of differentially expressed genes in Par (**L**), D-R (**M**), and P-R (**N**) cell lines, as well as for the 392 commonly regulated genes (**O**), with significance defined as *p* < 0.05 and |z-score| ≥ 2. The *x*-axis represents −log (*p*-value), while the *y*-axis represents the z-score, indicating activation (positive) or inhibition (negative). The size of each bubble corresponds to the number of genes regulated, and the color reflects the activation state (red for activated, blue for inhibited). Regulators are ranked based on statistical significance. All pathway enrichment and upstream regulator analyses were conducted using statistically significant differentially expressed genes (|FC| ≥ 1.5, *p* < 0.05, FDR < 0.1).

To better understand the molecular changes that may be responsible for mediating the differential transcriptomic profiles of D-R and P-R cells, we performed gene set enrichment analysis (GSEA) using differentially expressed genes identified in parental and chemotherapy-resistant (D-R and P-R) MDA-MB-231-ERβ cell lines following E2 treatment. Both early and late estrogen response pathways were enriched in all three cell lines, with normalized enrichment scores (NES) indicating strong activation of these pathways in parental cells (NES = 2.09 and 2.04 for early and late responses, respectively) (Figure 4A), D-R cells (NES = 1.50 and 1.57) (Figure 4B) and P-R cells (NES = 1.63 for both) (Figure 4C). Heatmaps were generated using the early and late E2 gene sets following vehicle and E2 treatment (Supplemental Figure S4A,B). Many other gene signatures were found to correlate with E2-regulated genes in the three different cell lines studied (Supplemental Table S2).

We next performed Enrichr-KG pathway analysis using all E2-regulated genes from parental, D-R, and P-R cell lines. While there is no directionality associated with this analysis, differentially expressed genes in parental cells were predominantly related to cell cycle control, DNA replication, p53 signaling, and cellular senescence (Figure 4D, Supplemental Table S3). In D-R cells, E2-regulated genes were most commonly related to multiple inflammatory, cytokine/chemokine, cell migration, and cell proliferation processes (Figure 4E, Supplemental Table S3). In P-R cells, nuclear receptor, glycosylation, cell migration, and differentiation were among the most enriched cellular processes following E2 treatment (Figure 4F, Supplemental Table S3). Using only the 392 genes that were commonly regulated by E2 in all three cell lines, the vitamin D receptor, glucocorticoid receptor, TGFβ, and extracellular matrix/fibrosis-related pathways were among the most abundant (Figure 4G and Supplemental Table S3).

Similarly, all E2-regulated gene sets from each cell line were interrogated via Ingenuity Pathway Analysis (IPA). In parental cells, E2 treatment potently suppressed numerous pathways related to cell cycle progression, DNA synthesis, and mitochondrial function while many fewer pathways were found to be induced such as granzyme A signaling, cell cycle checkpoint control and cellular senescence (Figure 4H, Supplemental Table S4). In D-R cells, only two pathways were significantly repressed by E2 and included RHOGDI signaling and antioxidant actions of vitamin C while many more pathways were induced including multiple inflammatory and immune-related pathways as well as cellular senescence (Figure 4I, Supplemental Table S4). In P-R cells, E2 was found to significantly regulate many fewer pathways which included the downregulation of glycation, HMGB1, macrophage, and EMT-related processes and the upregulation of neutrophil degranulation and other inflammatory signaling pathways (Figure 4J, Supplemental Table S4). Notably, several of these inflammatory and immune-related pathways in D-R and P-R cells included suppression of inflammatory cytokines such as IL-4, IL-10, and TNF. These cytokines are known to activate NFκB signaling and their suppression by E2 indicates that the downregulation of this pathway by ERβ is conserved in the setting of chemotherapy resistance (Supplemental Table S4).

Given that the anti-neoplastic activity of ERβ was maintained in the chemotherapy-resistant models, we next focused on the core set of transcriptional changes that were observed following E2 treatment of all three cell lines. Using this 392 gene signature, IPA identified only positively regulated pathways that included multiple immune and inflammatory processes, multiple growth factor/cytokine signaling pathways, glycosylation, and cytoskeletal/trafficking mechanisms (Figure 4K, Supplemental Table S4). Upstream regulator assessment of gene sets from individual cell lines revealed multiple estrogen receptors/regulatory factors and ligands. Using the list of 392 commonly regulated genes, a total of 97 upstream regulators met statistical significance with the two most highly significant factors being beta-estradiol and ERβ (Figure 4L–O, Supplemental Figure S5, Supplemental Table S5). Additional highly significant regulators included predicted activation of 4-hydroxy-tamoxifen, BRCA1, and dexamethasone. Predicted inhibited factors included TREM1, FOXA1, VCAN, and TREX1 (Figure 4L–O, Supplemental Figure S5, Supplemental Table S5).

As an additional point of comparison, we also performed IPA on the gene expression changes that were detected between D-R and P-R cells relative to parental controls in the absence of estrogen treatment. These pathways and biological processes associated with chemotherapy resistance in these MDA-MB-231 models are shown in Supplemental Figure S3C,D and Supplemental Table S6. Overall, these data have revealed the global transcriptional adaptations that occurred in MDA-MB-231 TNBC cells following acquired chemotherapy resistance and have highlighted the conserved biological pathways and cellular processes that are regulated by ERβ in chemotherapy-sensitive and -resistant TNBC. These conserved mechanisms are likely to be essential for the anti-neoplastic activity observed following ligand-mediated activation of ERβ.

### 3.5. Identification and Validation of ERβ-Mimicking Compounds

**Figure 5:**
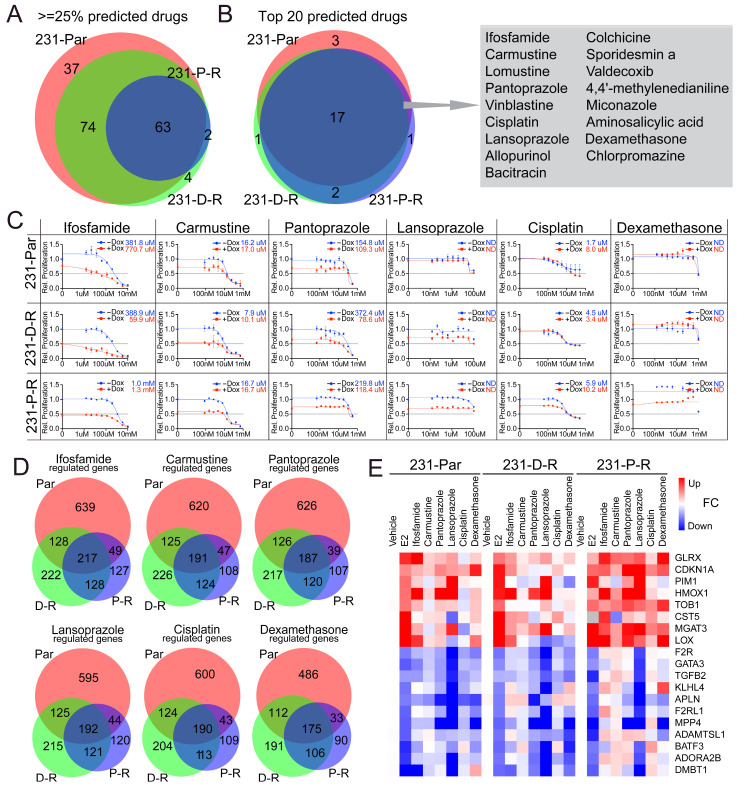
Alternative compounds that recapitulate the transcriptional effects of ERβ in chemotherapy-sensitive and -resistant MDA-MB-231 cells. (**A**,**B**) Venn diagrams depicting the overlap of compounds predicted to elicit similar transcriptomic profiles as that of E2 in each cell line according to the Mergeomics pipeline. (**C**) IC50 determinations for 6 of the top-ranking compounds in parental, D-R and P-R cell lines in the absence (blue line) and presence of ERβ expression (red line). (**D**) The differentially regulated transcripts of indicated compounds were retrieved from the Mergeomics database and were compared to the list of common E2-regulated genes identified in parental, D-R and P-R cells. A list of genes found to be similarly regulated by E2 and a given compound in the three cell lines was compiled and used to generate Venn diagrams. (**E**) Heatmaps depicting the results of RT-PCR analysis for indicated genes in parental, D-R and P-R cells following treatment with E2 or IC50 concentrations of the 6 indicated compounds for 24 h relative to vehicle-treated control cells. Data shown in panels (**C**,**E**) are representative of 8 and 3 technical replicates, respectively, that were derived from one of three independent experiments.

To identify alternative therapeutic compounds capable of eliciting similar transcriptomic responses, and thus perhaps capable of recapitulating ERβ’s tumor suppressive activity even in the absence of ERβ expression, we used the publicly available PharmOmics pipeline (Mergeomics) [23,26] to independently analyze the E2-regulated gene sets from each of our cell line models (parental, D-R, and P-R). Significantly upregulated and downregulated genes in response to E2 treatment were input separately, with directionality preserved during analysis. The comparison of the compounds identified for each model system revealed many molecules that were predicted to elicit similar gene expression profiles to that of E2, of which 63 compounds were identified in the top 25% of all three cell lines (Figure 5A). The predicted drug signatures for each model were then compared to identify compounds consistently overlapping across all three models (Figure 5A,B). Of the top 20 compounds identified for each cell line, 17 were common between all models (Figure 5B). Then, 6 of these 17 were prioritized based on their rank scores and significance, as well as the drug class to which they belonged, including ifosfamide, carmustine, pantoprazole, lansoprazole, cisplatin, and dexamethasone.

The dose response curves of these six drugs demonstrated highly similar efficacy across parental and chemotherapy-resistant models, with little difference in IC50 values between the three cell lines (Figure 5C). The IC50s were also very similar regardless of ERβ expression, supporting the idea that these drugs function in an ERβ-independent manner. From a gene expression perspective, approximately half of all ERβ-regulated genes in the parental, D-R and P-R cells were also regulated by each of these compounds in the cell models where they were studied. Impressively, the distribution of unique and commonly regulated genes was found to be highly consistent among all drugs (Figure 5D, Supplemental Figure S6, Supplemental Table S7). To experimentally validate that these 6 compounds could partially recapitulate the effects of ERβ on the transcriptome, MDA-MB-231-ERβ parental, D-R, and P-R cells were treated with the IC50 concentrations identified for each compound, or E2, for 24 h. RT-PCR was then performed on a randomly selected sub-set of genes identified to be regulated by E2 in our RNAseq datasets. As shown in Figure 5E, the majority of genes regulated by E2 were similarly regulated by the 6 compounds, particularly in the parental and D-R models. Together, these results demonstrate that transcriptional profiling can identify candidate compounds that functionally converge with ERβ-regulated gene networks. While these compounds are not direct ERβ ligands, their ability to induce overlapping gene expression signatures highlights their potential to mimic the effects of ERβ, even in triple-negative breast cancer cells that do not express ERβ.

## 4. Discussion

We have previously demonstrated that ERβ is expressed in approximately 20% of treatment-naïve tumors and elicits significant anti-neoplastic activity in multiple cell line models of TNBC [11,12,15,16,17,27]. To determine the potential relevance of ERβ-targeted therapies in chemotherapy-resistant TNBC, we generated multiple models of doxorubicin and paclitaxel resistant cells, which were derived from doxycycline-inducible ERβ expressing models, and subsequently assessed their ERβ responsiveness.

As a first step, we demonstrated that E2 and LY, an ERβ agonist, retain the ability to inhibit proliferation, invasion and migration of chemotherapy-resistant TNBC cells in an ERβ dependent manner. Previous findings from our group identified the involvement of NFκB signaling and EZH2 function as critical components of ERβ activity. Specifically, we reported that ERβ suppressed NFκB signaling through the recruitment and repurposing of EZH2 at NFκB binding sites throughout the genome to silence the expression of NFκB -regulated genes [12]. Increased NFκB pathway activity is reported to induce resistance to multiple chemotherapy agents in TNBC [28] and is classically upregulated in chemotherapy-resistant tumors [29]. In the present study, we have confirmed several factors/pathways known to involve NFκB, such as IL-4, IL-10, TNF, and acute phase response signaling, to be enriched in D-R and P-R cell lines indicating that NFκB signaling is upregulated in the setting of chemotherapy resistance. These data also demonstrate that suppression of this oncogenic signaling pathway by ERβ remains intact in the setting of chemotherapy resistance and likely contributes to the anti-neoplastic activity of ERβ in this setting.

Building upon the transcriptomic analyses of the chemotherapy-sensitive and -resistant cell lines, we identified a minimal gene expression profile associated with the anti-neoplastic activity of ERβ. Specifically, 392 transcripts, or only about 1/3 of all ERβ-regulated genes in a given cell line, were found to be commonly regulated across all of the models studied. We surmise that this gene list contains the critical ERβ effector genes required to suppress proliferation, invasion, and migration. Pathway analysis of this minimal gene list revealed altered activity of pathways known to drive cancer progression and resistance, including TGFβ signaling, BMP signaling, extracellular matrix remodeling, nuclear receptor pathways, and cytokine modulation. The TGFβ signaling pathway, which plays a dual role in tumor suppression and progression, was significantly enriched, indicating a potential mechanistic role for ERβ in modulating TGFβ activity, consistent with previous findings on ERβ-mediated suppression of TGFβ signaling [17]. Additionally, alterations in extracellular structure organization, extracellular matrix (ECM) organization, and cytoskeleton regulation following E2 treatment highlight important roles for ERβ in modulating cell adhesion, migration, and metastatic potential in TNBC. Moreover, the pathway analysis highlighted metabolic alterations, including glycosylation, glycoprotein biosynthesis, lipid metabolic regulation, and glucose and energy metabolism, indicating a shift in cancer cell metabolism following E2-induced ERβ activation.

The predicted upstream regulators highlighted key regulatory mechanisms that were conserved across all cell lines in response to E2 treatment including hormone signaling, immune response, cell cycle control, and stress adaptation. β-estradiol (E2) and *ESR2* (ERβ) were the strongest activated regulators identified, reinforcing ERβ’s direct role in transcriptional control of key tumor suppressive genes involved in TGFβ signaling, ECM remodeling, metabolic shifts, and cytokine modulation. The activation of nuclear receptor modulators, including dexamethasone and tretinoin, and cytokine and inflammatory signaling, such as STAT3, IFNG, and IL6, along with inhibition of TREM1, suggests the regulation of inflammatory and immune responses [30,31,32,33] that are also mediated by ERβ in response to E2. ECM and cytoskeletal remodeling were key regulatory processes conserved across cell lines with BMP4 and KLF6 activation reinforcing TGFβ signaling in ECM organization [34]. VCAN, a major ECM component associated with tumor progression, was inhibited, while CDH11 was activated, highlighting ERβ’s ability to modulate cell adhesion and migration [35,36]. Significant enrichment in metabolic and oxidative stress-related pathways, including AMPK, FOXO1, and FOXO3, were also found, suggesting the involvement of ERβ in metabolic adaptation [37].

While these results are promising for the utility of ERβ agonists in chemotherapy-resistant TNBC, the majority of recurrent and metastatic TN tumors are ERβ negative. Thus, the identification of alternative mechanisms by which to recapitulate the effects of ERβ-targeted therapies, independent of ERβ expression, may have a profound clinical impact. Towards this goal, we utilized the Mergeomics pipeline and associated datasets to identify compounds which have been reported to elicit similar gene expression profiles as that of ERβ. These analyses revealed 20 drugs that confidently met these criteria. Six of these drugs were prioritized based on drug class and mode of action and were subsequently shown to elicit anti-proliferative activity in chemotherapy-sensitive and -resistant cell lines in an ERβ-independent manner. Importantly, we were able to confirm that these drugs resulted in altered expression of known ERβ target genes in ERβ negative cells, effects that were nearly identical to that of E2 in ERβ-positive cells. Interestingly, previous studies by our group have implicated a number of these commonly regulated genes, and their associated pathways, as essential mediators of ERβ activity. Specifically, we have shown that ligand-mediated activation of ERβ results in robust induction of CST5 expression and subsequent inhibition of TGFβ signaling [27]. Here, CST5 was shown to be induced by these 6 compounds, to levels similar to that of E2, across sensitive and resistant cell lines while TGFβ2, the ligand responsible for activating canonical TGFβ signaling, was repressed. These results suggest that these alternative treatments can at least partially recapitulate the effects of ERβ in TNBC and indicate that strategies leading to CST5 induction, and repression of TGFβ signaling, are viable alternative approaches for the treatment of advanced TNBC regardless of ERβ expression.

## 5. Conclusions

In summary, our findings provide critical insights into the role of ERβ as a tumor suppressor in TNBC, particularly in the context of chemotherapy resistance. Through a combination of transcriptomic profiling, pathway analysis, and functional assays, we demonstrated that ERβ retains its anti-neoplastic activity in both chemotherapy-sensitive and chemotherapy-resistant TNBC cell models. Remarkably, resistant cells exhibited increased sensitivity to ERβ-targeted therapies, suggesting that ERβ agonists may have activity even in advanced and refractory disease settings for a sub-set of patients. This highlights the potential of leveraging ERβ expression/activation as a therapeutic approach for the management of chemotherapy-resistant TNBC.

## Data Availability

The data generated in this study are publicly available in Gene Expression Omnibus (GEO) at GSE300447.

## Supplementary Materials

The following supporting information can be downloaded at https://www.mdpi.com/article/10.3390/cancers17132132/s1, Figure S1: Generation of chemotherapy-resistant MDA-MB-468-ERβ cells. Figure S2: Karyotyping analysis of MDA-MB-231-ERβ parental and chemotherapy-resistant cell lines. Figure S3: Differential gene expression profiling of chemotherapy-resistant MDA-MB-231-ERβ cell lines relative to parental controls. Figure S4: Expression patterns of early and late estrogen response genes in parental and chemotherapy-resistant MDA-MB-231-ERβ cell lines. Figure S5: Upstream regulator analysis of parental and chemotherapy-resistant MDA-MB-231-ERβ cell lines. Figure S6: Overlap of compound-regulated genes with the 392 E2 gene signature. Table S1: Primer sequences for real-time quantitative PCR. Table S2: Gene Set Enrichment Analysis (GSEA) of E2-regulated genes in parental and resistant MDA-MB-231-ERβ cell lines. Table S3: Enrichr-KG pathway analysis of E2-regulated genes in parental and resistant MDA-MB-231-ERβ cell lines, and commonly regulated genes across all models. Table S4: Ingenuity Pathway Analysis (IPA) of E2-regulated genes in parental and resistant MDA-MB-231-ERβ cell lines, and commonly regulated genes across all models. Table S5: Upstream regulator analysis of E2-regulated genes in parental and resistant MDA-MB-231-ERβ cell lines, and commonly regulated genes across all models. Table S6: Ingenuity Pathway Analysis (IPA) and upstream regulator analysis of differentially expressed genes in D-R and P-R cells relative to parental controls under vehicle treatment conditions. Table S7: Overlap between Mergeomics-predicted compound-regulated gene sets and the 392 commonly regulated genes across parental and resistant models.
