# Supplementary material for: Anti-Neoplastic Activity of Estrogen Receptor Beta in Chemoresistant Triple-Negative Breast Cancer
Source: Cancers (Basel). 2025 Jun 25;17(13):2132. doi: 10.3390/cancers17132132 (PMC12249222; doi:10.3390/cancers17132132)
Supplement: Supplementary file 1 [file cancers-17-02132-s001.zip › cancers-3687988-supplementary/Supplemental Figures.pdf]

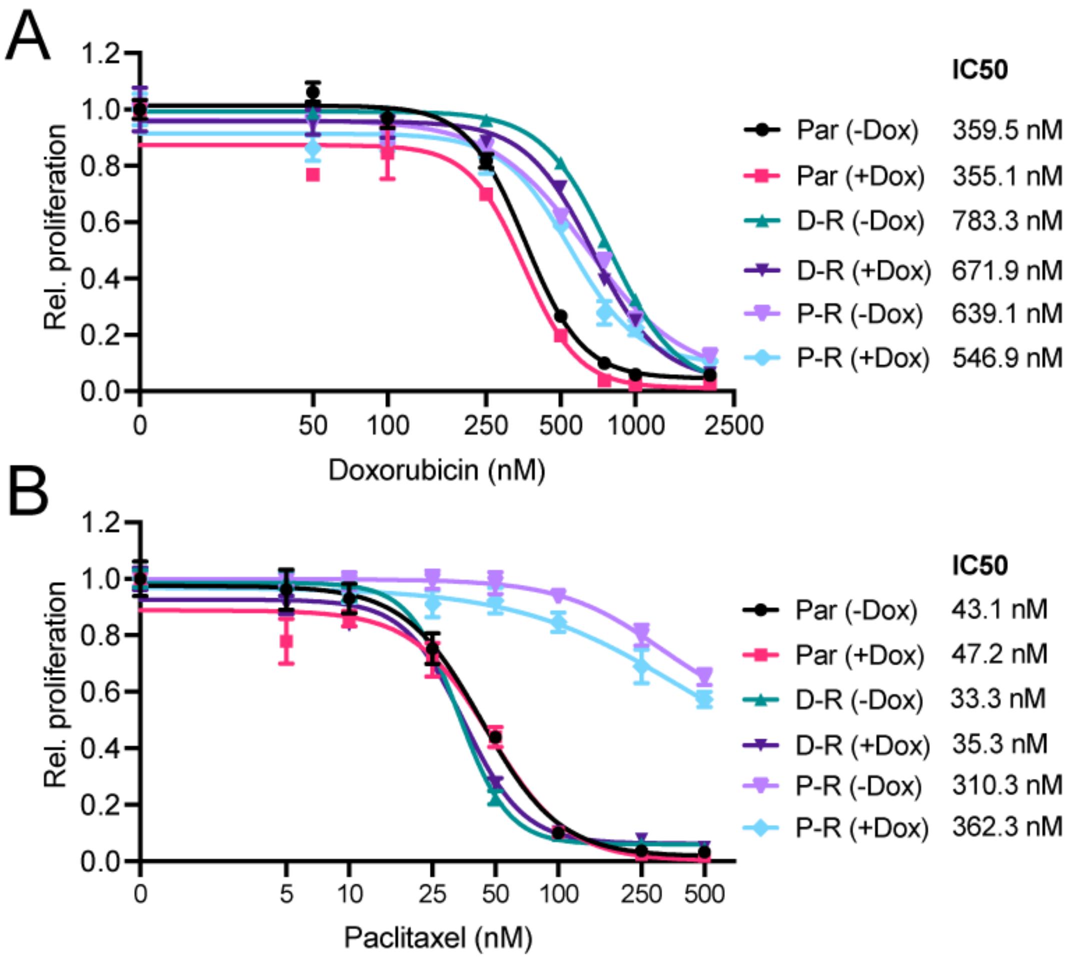

Supplemental Figure S1. Generation of chemotherapy-resistant MDA-MB-468-ERβ cells. Proliferative response of parental (Par), doxorubicin-resistant (D-R), and paclitaxel-resistant (P-R) MDA-MB-468-ERβ cells to doxorubicin (A) or paclitaxel (B). IC50 values are indicated. The graphs are representative of 8 technical replicates derived from one of three independent experiments.

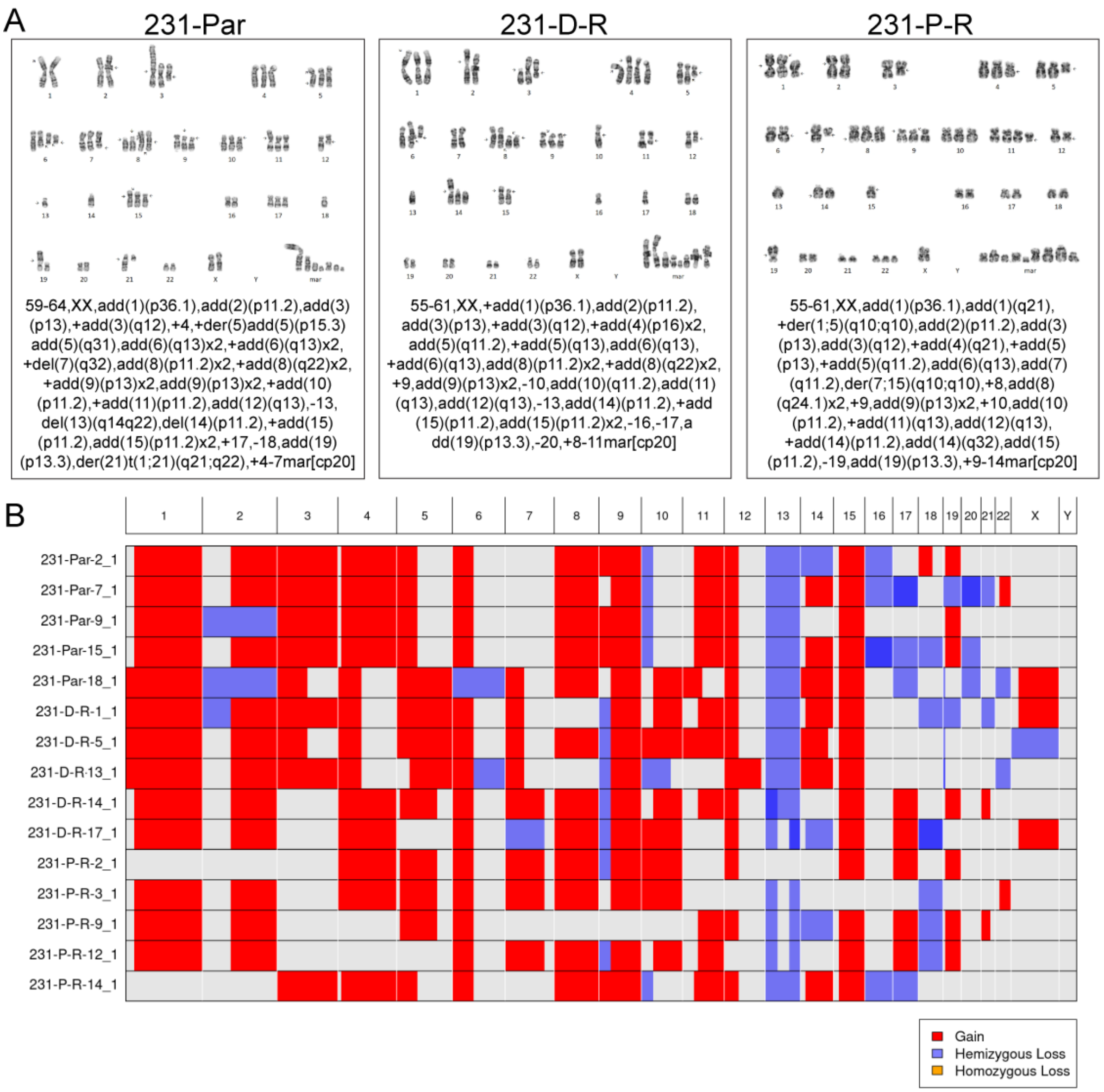

Supplemental Figure S2. Karyotyping analysis of MDA-MB-231-ERβ parental and chemotherapy-resistant cell lines. A) Representative karyotypes of parental, D-R, and P-R cells, showing complex metaphases with similar ploidy levels and multiple chromosomal abnormalities. B) Summary of key chromosomal alterations observed in 5 representative cells out of 20 total cells analyzed for Par, D-R, and P-R models, generated using CytoConverter.

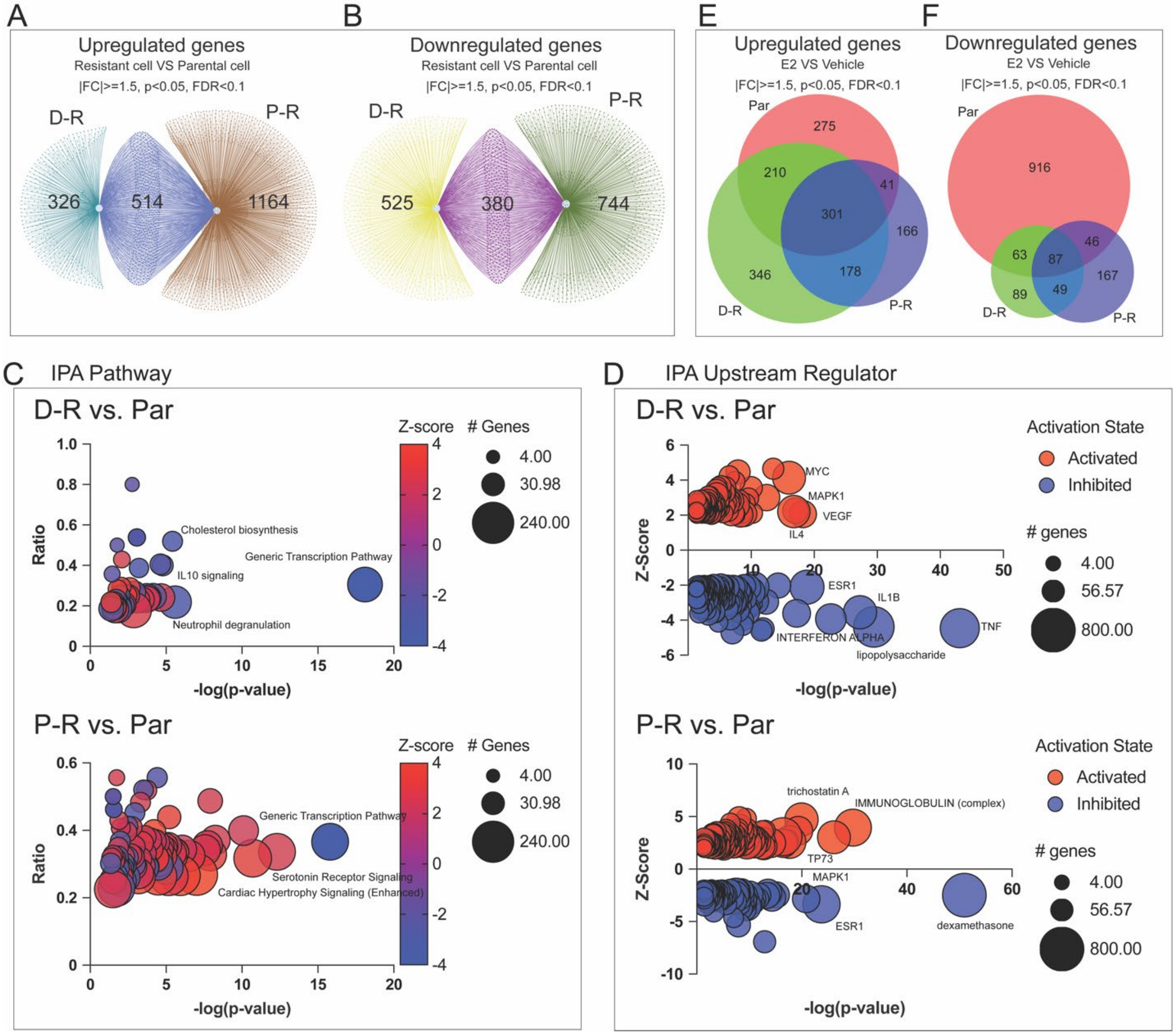

Supplemental Figure S3. Differential gene expression profiling of chemotherapy-resistant MDA-MB-231-ERβ cell lines relative to parental controls. (A) Upregulated and (B) downregulated genes in D-R and P-R cells compared to parental (Par) cells under vehicle-treated conditions. IPA of significantly dysregulated genes identified in D-R and P-R cells compared to parental cells, showing altered canonical pathways (C) and predicted upstream regulators (D) (z-score threshold |┬ 2|, $p < 0.05$). Venn diagrams depicting overlap of upregulated € and downregulated (F) genes in parental, D-R, and P-R cells in response to 24-hour treatment with 1 nM E2 compared to vehicle control. The Venn diagrams were generated using E Venn and BioVenn.

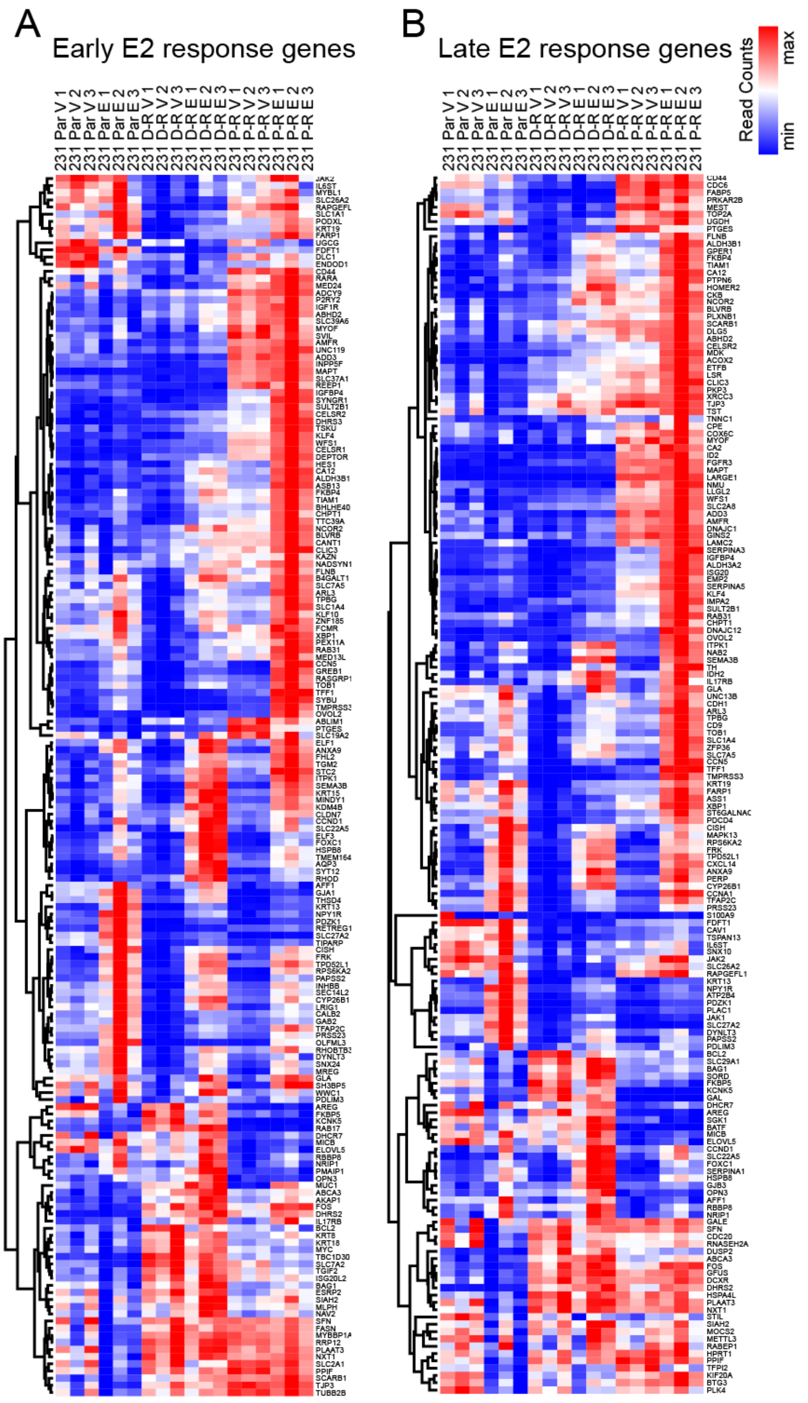

Supplemental Figure S4. Expression patterns of early and late estrogen response genes in parental and chemotherapy-resistant MDA-MB-231-ERβ cell lines. A) Heatmap of early estrogen response genes across parental, D-R, and P-R MDA-MB-231-ERβ cells following 24-hour treatment with vehicle (V) or 1 nM E2 (E). B) Heatmap of late E2 response genes under the same treatment conditions. Gene expression was analyzed using RNA-seq, and hierarchical clustering was applied to visualize differences in transcriptional responses. The heatmaps were generated using Morpheus.

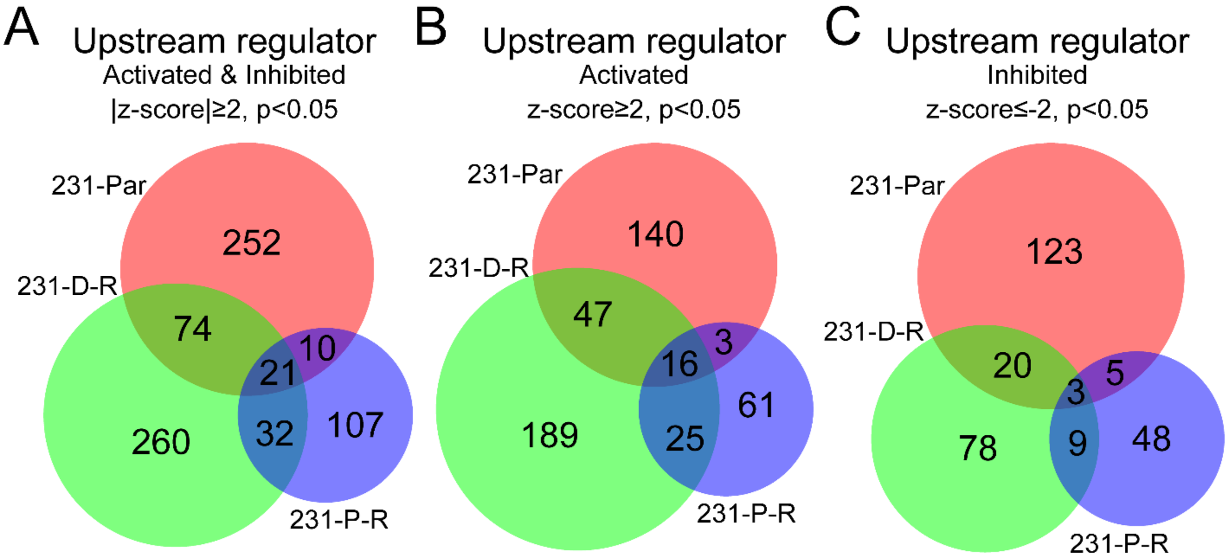

Supplemental Figure S5. Upstream regulator analysis of parental and chemotherapy-resistant MDA-MB-231-ERβ cell lines. . Venn diagrams depicting overlap of all IPA predicted upstream regulators (A), or only those predicted to be activated (B) or inhibited (C), in parental, D-R, and P-R cells following 1 nM E2 treatment. Significance we considered based on z-score ≥ 2, $p < 0.05$. Venn diagrams were generated using BioVenn.

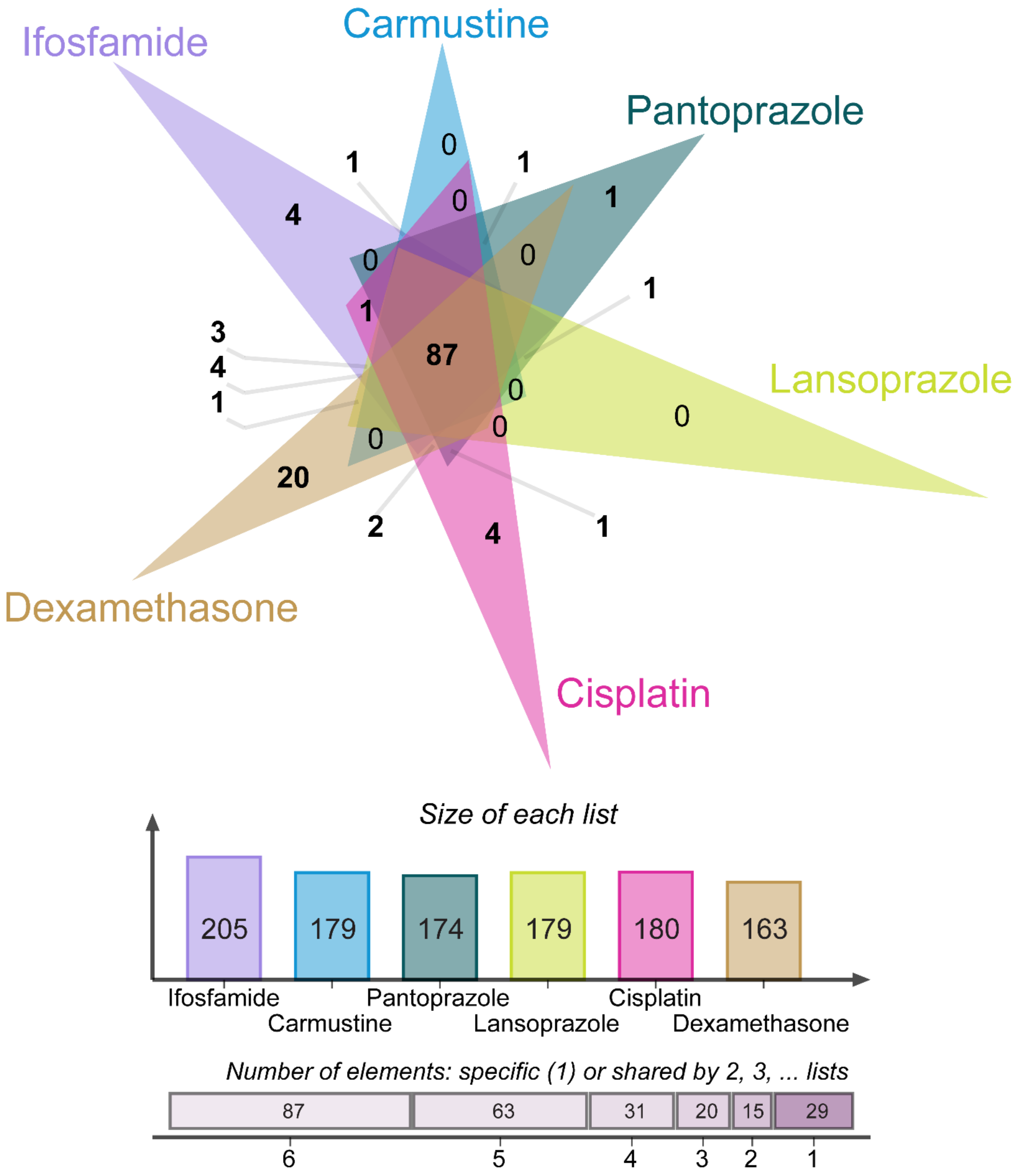

Supplemental Figure S6. Overlap of compound-regulated genes with the 392 E2 gene signature. Venn diagram depicting the overlap of genes known to be differentially expressed in response to the six top-ranking compounds (ifosfamide, carmustine, pantoprazole, lansoprazole, cisplatin, and dexamethasone) with the 392 genes commonly regulated by E2 treatment in parental and chemoresistant MDA-MB-231 cell lines.
